# Surface-based multimodal protein–ligand binding affinity prediction

**DOI:** 10.1093/bioinformatics/btae413

**Published:** 2024-06-21

**Authors:** Shiyu Xu, Lian Shen, Menglong Zhang, Changzhi Jiang, Xinyi Zhang, Yanni Xu, Juan Liu, Xiangrong Liu

**Affiliations:** National Institute for Data Science in Health and Medicine, Xiamen University, Xiamen 361005, China; Department of Computer Science and Technology, Xiamen University, Xiamen 361005, China; Department of Computer Science and Technology, Xiamen University, Xiamen 361005, China; Department of Computer Science and Technology, Xiamen University, Xiamen 361005, China; Department of Computer Science and Technology, Xiamen University, Xiamen 361005, China; Department of Computer Science and Technology, Xiamen University, Xiamen 361005, China; Pen-Tung Sah Institute of Micro-Nano Science and Technology, Xiamen University, Xiamen 361005, China; National Institute for Data Science in Health and Medicine, Xiamen University, Xiamen 361005, China; Department of Computer Science and Technology, Xiamen University, Xiamen 361005, China; Xiamen Key Laboratory of Intelligent Storage and Computing, Xiamen University, Xiamen 361005, China

## Abstract

**Motivation:**

In the field of drug discovery, accurately and effectively predicting the binding affinity between proteins and ligands is crucial for drug screening and optimization. However, current research primarily utilizes representations based on sequence or structure to predict protein–ligand binding affinity, with relatively less study on protein surface information, which is crucial for protein–ligand interactions. Moreover, when dealing with multimodal information of proteins, traditional approaches typically concatenate features from different modalities in a straightforward manner without considering the heterogeneity among them, which results in an inability to effectively exploit the complementary between modalities.

**Results:**

We introduce a novel multimodal feature extraction (MFE) framework that, for the first time, incorporates information from protein surfaces, 3D structures, and sequences, and uses cross-attention mechanism for feature alignment between different modalities. Experimental results show that our method achieves state-of-the-art performance in predicting protein–ligand binding affinity. Furthermore, we conduct ablation studies that demonstrate the effectiveness and necessity of protein surface information and multimodal feature alignment within the framework.

**Availability and implementation:**

The source code and data are available at https://github.com/Sultans0fSwing/MFE.

## 1 Introduction

As a crucial stage in drug discovery, predicting protein–ligand binding affinity has been intensively studied for a long time (Sousa *et al.* 2006, Jacob and Vert 2008), which is of great importance for efficient and accurate drug screening (Li *et al.* 2021). Traditional computer-aided drug discovery tools use scoring functions (SF) to estimate protein–ligand binding affinity roughly (Jacob and Vert 2008) but with lower accuracy. Molecular dynamics simulation methods can provide more accurate estimates of binding affinity (Deng and Roux 2009), but are often costly and time-consuming.

With the significant development of computing technology and the increasing abundance of large-scale biological data, deep learning-based methods have shown great potential in the field of protein–ligand binding affinity prediction. For example, methods such as DeepDTA (Öztürk *et al.* 2018), DeepDTAF (Wang *et al.* 2021a), and DeepAffinity (Karimi *et al.* 2019) use sequence information of proteins and ligands to predict the binding affinity between them. However, existing sequence-based methods lack 3D structure information, prompting researchers to leverage 3D grid data representations of proteins’ geometric structures and use 3D Convolutional Neural Networks (3D CNNs) for affinity prediction to learn the geometric features of proteins (Ragoza *et al.* 2017, Jiménez *et al.* 2018). With the recent development of Graph Neural Networks (GNNs), the advantages of using graphs to represent proteins and ligands are gradually being highlighted in deep learning-based models (Lim *et al.* 2021), such as graph convolutional network (GCN) (Townshend *et al.* 2020), graph attention network (GAT) (Yuan *et al.* 2021) and graph isomorphism network (GIN) (Xu *et al.* 2019). But directly applying GNNs to process the 3D structures of proteins falls short in adequately capturing their geometric information. Therefore, GBPNet (Aykent and Xia 2022) incorporates direction vectors as node and edge features and uses an SO(3) equivariant message passing network to learn the proteins’ geometric representations for predicting protein–ligand binding affinity.

However, these methods do not take into account the important role that molecular surface information plays in protein–ligand interactions. Molecular surface, a high-level representation of protein structure, exhibits patterns of chemical and geometric features that serve as fingerprints for the protein’s modes of interaction with other biomolecules (Gainza *et al.* 2020). Therefore, some studies begin to use protein surface information to predict protein–ligand binding affinity. For example, MaSIF (Gainza *et al.* 2020) pioneers surface-based geometric deep learning to solve protein interaction related tasks. It computes the geometric and chemical features of each vertex in the surface mesh and utilizes geometric deep neural networks to learn interaction fingerprints in protein molecular surfaces. In HOLOPROT (Somnath *et al.* 2021), researchers use the triangulation software MSMS (Connolly 1983, Sanner *et al.* 1996) to generate a mesh of the protein surface, with each surface node pointing to the corresponding amino acid residue through directed edges, and use a multi-scale message passing network to learn protein representations at different scales for protein–ligand binding affinity prediction. Unfortunately, adopting meshes to represent protein surfaces encounters several challenges. The primary issues include the necessity to pre-compute input features and mesh connectivities, which substantially increases computational time and demands significant memory resources. To avoid these problems, dMaSIF (Sverrisson *et al.* 2021) generates a point cloud representation of the protein surface by inputting only atomic coordinates and types, learns task-specific geometric and chemical features on the surface point cloud, and finally applies a new convolutional operator that approximates geodesic coordinates in the tangent space.

Although the previously mentioned research approaches have yielded promising outcomes, they mainly focus on singular modal data, overlooking the multimodal information of proteins. In recent years, some researchers (Ngo and Hy 2023, Zhang *et al.* 2023) have begun to realize the limitations of single modal information and have instead tried to combine sequence and structure information. However, the feature embedding of different modalities are initially located in different subspaces. This direct information fusion method, such as concatenating the embeddings of various modalities (Hu *et al.* 2023), often ignores the heterogeneity between different modalities and cannot fully exploit the complementarity between different modalities.

In this paper, we propose a novel multimodal feature extraction (MFE) framework that, for the first time, incorporates information from protein surfaces, 3D structures and sequences. Specifically, we design two main components: a protein feature extraction module, and a multimodal feature alignment module. The protein feature extraction module is used to extract the initial embeddings from protein surface, structure and sequence information. In the multimodal feature alignment module, we use cross-attention mechanism to achieve feature alignment between protein structure, sequence embedding and surface embedding to obtain unified and information-rich feature embedding. Compared with current state-of-the-art methods, the proposed framework achieves optimal results on the protein–ligand binding affinity prediction task.

Our contributions can be summarized as follows:

- We propose a novel framework to extract the initial embeddings from protein surface, structure and sequence, and efficiently align and fuse three different modalities of initial embeddings.
- Our proposed framework can make the model interpretable. The attention mechanism can make the model pay attention to the most relevant parts of different modalities, which is beneficial for the feature alignment and fusion.
- Our evaluation of the model using the PDBbind dataset demonstrates that our framework outperforms current state-of-the-art methods in predicting protein–ligand binding affinity.

## 2 Materials and methods

**Figure 1.**
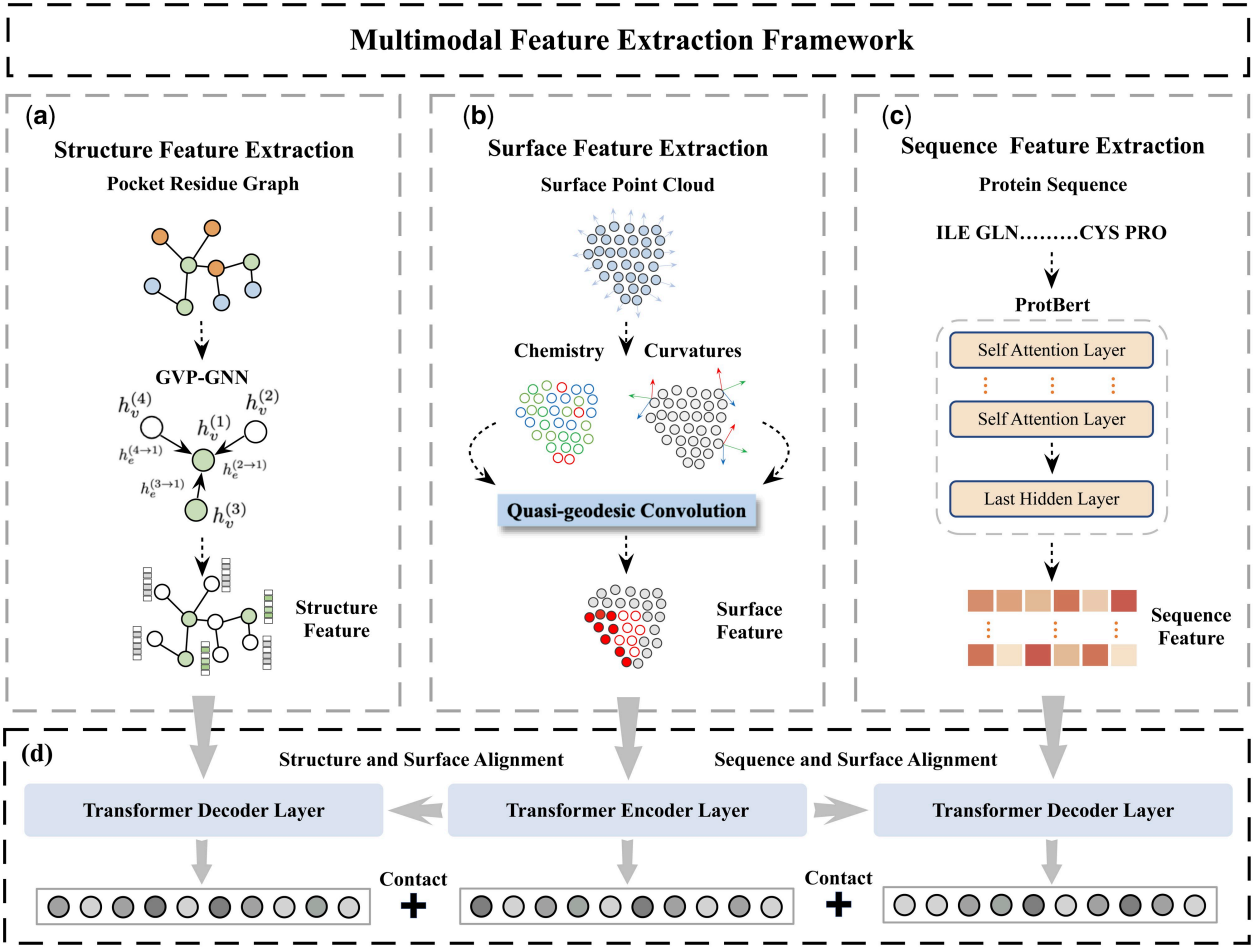
Illustration of the MFE framework. (a)–(c) Protein feature extraction module. (d) Multimodal feature alignment module.

In this section, we first introduce the MFE framework in detail, which consists of protein feature extraction module and multimodal feature alignment module. Then we introduce its application on the protein–ligand binding affinity prediction task. The main framework of the model is shown in Fig. 1.

### 2.1 Protein feature extraction module

The protein-binding pocket refers to the protein surface or interior cavity directly binding to the ligand, and plays important role in determining the protein–ligand binding affinity (Jin *et al.* 2023). The amino acid residues around it determine its physical and chemical properties and functions, and these properties are critical to the specific interaction between the protein and the ligand. Therefore, in protein–ligand binding affinity prediction, using protein pocket information can help predict the binding ability between protein and ligand more accurately. However, if only the surface and structure information of the protein pocket are considered, the global information of the protein is ignored. From a microscopic point of view, a protein is essentially a long sequence of amino acids, and this sequence undergoes changes such as folding in three dimensional space to form a complete protein structure (Zhang *et al.* 2023). Therefore, we use the complete amino acid sequence to represent the global information of the protein.

### 2.2 Surface feature extraction

Protein molecular surfaces carry important geometric and chemical information indicative of the way they interact with other molecules (Sverrisson *et al.* 2021). Here we use the sampling algorithm proposed in dMaSIF (Sverrisson *et al.* 2021) to calculate and generate protein surfaces on-the-fly from the underlying atomic point cloud. Specifically, we first input an atomic cloud containing 22 atom types, and sampled to obtain an oriented point cloud representation of the protein surface. We then selected the 512 surface points closest to the center of the ligand as surface pockets.

**Chemical feature.** For each selected point, instead of using traditional protein chemical descriptors such as electrostatic charge or hydrophilicity, we select the 16 nearest atom centers along with their atom types to compute a vector of chemical features through multilayer perceptrons. As dMaSIF (Sverrisson *et al.* 2021) demonstrates, chemical properties such as Poisson-Boltzmann electrostatics can be simulated from primitive chemical features such as atom type distributions.

**Geometric feature.** To characterize the geometry of the surface point cloud, we compute the Mean curvature and Gaussian curvature as geometric features for each point, and the obtained geometric features and chemical features are spliced together as a complete feature vector.

**Quasi-geodesic convolutions.** Here we use quasi-geodesic convolution layers to obtain the final scalar embedding of the surface point. Quasi-geodesic convolution is a convolution operation applied to protein surface-oriented point clouds and is able to learn problem-specific features directly from the surface point cloud of proteins instead of relying on precomputed descriptors. Quasi-geodesic convolution is invariant to 3D rotations and translations, meaning the model can make predictions based on the local chemical and geometric properties of the protein surface, independent of the protein’s specific location in space.

### 2.3 Structure feature extraction

As a biological macromolecule, protein has a complex structure. It is usually represented by a residue graph, where nodes represent amino acid residues and edges represent interactions between residues, such as Hydrogen bonds, hydrophobic interactions, or spatial proximity relationships. At present, Graph Neural Networks (GNNs) are widely used to capture the features of protein residue graphs. However, GNNs mainly focuses on the topological relationship between nodes in the graph, rather than the specific positions and directions of these nodes in 3D space. The Geometric Vector Perceptron (GVP) (Jing *et al.* 2021) addresses this limitation by integrating not only the topological features but also the spatial orientation and position of nodes. The input to the GVP model consists of a tuple (*s*, *V*), where $s\in R^{n}$ represents scalar features designed to ensure rotational invariance of molecules, and $V\in R^{v\times 3}$ comprises vector features that indicate the absolute directions of each node. These vector features can be propagated directly in the standardized global coordinate system of the entire structure, making it easier for GNNs to access the global geometric properties of the structure (Hu *et al.* 2023). We use the GVP-GNN to extract 3D structural features of proteins. All nodes and edges in this GNN are represented using tuples containing scalars and vectors, enabling efficient representation of 3D structures of protein through geometric and relational reasoning (Zheng *et al.* 2023). The GVP-GNN updates node embeddings through message propagation steps according to:

(1) $$m_{ij}=\mathrm{GVPs}(\mathrm{concat}(h_{v}^{\left(j\right)},h_{e}^{\left(j\to i\right)})),$$

(2) $$h_{v}^{\left(i\right)}=\mathrm{LayerNorm}(h_{v}^{\left(i\right)}+\frac{1}{k\prime }D\mathrm{ropout}(\sum_{j:e_{j\to i}}^{\in \varepsilon }m_{ij})),$$

where $h_{v}^{\left(i\right)}$ represents the embedding of node *i*, while $h_{e}^{\left(j\to i\right)}$ denotes the embedding of the edge (j→i). *m_ij_* represents the message passed by node *j* to node *i* calculated by three GVP layers, and k′ is the number of incoming messages.

### 2.4 Sequence feature extraction

The properties and functions of a protein are determined by its amino acid sequence and the way it folds in 3D space. The 1D amino acid sequence of a protein can be regarded as a special “biological language” that has natural similarities with natural language. Many methods based on Natural Language Processing (NLP) can be directly extended to process amino acid sequences. Here, we process the input sequence through ProtBert (Elnaggar *et al.* 2022) to obtain the initial sequence embedding of our model. ProtBert is a pretrained model on protein sequences using a masked language modeling (MLM) objective. It was pretrained on the public dataset Uniref100 and can be used for protein sequence analysis and predict. It should be noted that ProtBert is only used to obtain the initial sequence embedding, and its network weights are not involved in subsequent multimodal training.

### 2.5 Multimodal feature alignment module

The effective fusion of protein multimodality is affected by the heterogeneity of its different modalities and scale differences. Commonly used techniques such as concatenating the embedding often ignore the heterogeneity between different modalities when processing protein data, which may lead to the loss of modality-specific features (Hu *et al.* 2023). The Transformer architecture provides an effective solution to this problem. It is capable of processing sequential data and capturing long-distance dependencies through the self-attention mechanism, as well as learning the data between different modalities through the cross-attention layer, achieving effective alignment and fusion of features.

Specifically, we first use the Transformer Encoder to process the embedding of the protein surface, utilizing the self-attention mechanism to deeply learn the details and characteristics of the protein surface. Subsequently, through the Transformer Decoder, we globally align the structure and sequence embeddings of the protein with the new surface embedding obtained after processing by the Encoder. This not only retains the unique information and complementarity of each modality but also promotes effective communication between different modalities. Finally, we apply average pooling to the new embeddings of the surface, structure, and sequence separately. These pooled embeddings are then merged to create a unified and context-rich feature representation. The application of this method not only improves the accuracy of predictions but also offers a new perspective for understanding the complexity of proteins.

### 2.6 Protein–ligand binding affinity prediction

We evaluate our framework on the task of protein–ligand binding affinity prediction. We treat small molecule ligands as 2D graphs, with atoms in the ligand corresponding to nodes in the graph, and covalent bonds between atoms corresponding to edges in the graph. AttentiveFP (Xiong *et al.* 2020) is used to learn the representation of ligands. This method can use the attention mechanism to capture the complex interactions between atoms. It has a certain interpretability and achieves good performance on a variety of tasks.

In order to better study the interaction between protein and ligand, we construct a heterogeneous graph $G=(V_{l},V_{p},E)$, where *V_l_* represents the set of atoms in the ligand molecule, *V_p_* represents the set of atoms in the protein. If the distance between ligand atoms $V_{l}^{i}$ and protein atoms $V_{p}^{j}$ is less than the cutoff distance *c *=* *5 Å, we set *e_ij_* = 1. It is worth noting that there are no edges in the same atom set. We construct the global embedding of the entire heterogeneous graph by performing message passing on two sets of nodes respectively, and utilizing edge-level pooling and multilayer perceptron.

At the end of the model, we connect the multimodal feature embeddings of the protein, the embedding of the ligand graph, and the embedding of the heterogeneous graph and pass them to the multilayer perceptron for affinity prediction.

The model uses Mean Squared Error (MSE) as the loss function, and uses the Adam Optimizer to optimize parameters:

(3) $$\mathrm{MSE}=\frac{1}{N}\sum_{i=1}^{N}{\left(y_{i}-\hat{y_{i}}\right)}^{2},$$

where N represents the number of sample pairs contained in the dataset.

## 3 Results and discussion

### 3.1 Dataset

The PDBbind dataset (version 2016) (Wang *et al.* 2005) contains biomolecular complexes from the Protein Data Bank (Berman *et al.* 2000) and their experimentally measured binding affinity data, and is often used for protein–ligand binding affinity prediction tasks. The dataset is divided into 3 subsets: general set, refined set and core set. The general set contains 9228 protein–ligand complexes, but the data quality is uneven; the refined set contains 4057 complexes with higher data quality; the core set contains 285 carefully selected the highest quality complexes and commonly used as a test set for protein–ligand binding affinity prediction tasks. Here we directly use the core set as the test set, and the complexes in the core set are removed from the general set and refined set to avoid data leakage. And 1000 complexes were randomly selected from the refined set as the validation set, and then the remaining complexes were merged into the general set as the training set.

### 3.2 Baseline

For evaluating the overall performance of our model, we compare the Multimodal Feature Extraction framework against some wide-ranging popular or state-of-the-art baselines. The baselines discussed here can be categorized into four distinct groups. The first group consists of sequence-based methods, exemplified by DeepDTA (Öztürk *et al.* 2018). The second group encompasses structure-based methods, represented by Pafnucy (Stepniewska-Dziubinska *et al.* 2018), OnionNet (Zheng *et al.* 2019), and CurvAGN (Wu *et al.* 2023). The third group focuses on surface-based methods, with dMaSIF (Sverrisson *et al.* 2021) being a notable example. Lastly, the fourth group is comprised of multimodal methods, which include DPLA (Wang *et al.* 2021b) and HaPPy (Zhang *et al.* 2023).

### 3.3 Error evaluation metrics

We use the root mean square error (RMSE) and the mean absolute error (MAE) to evaluate the prediction error of the model. In addition, we use two indicators, the standard deviation (SD) and the Pearson correlation coefficient (R), to measure the correlation between the predicted values and the true values.

### 3.4 Experimental results

Table 1 shows the results of our and other baseline models on the protein–ligand binding affinity prediction task. All models use the same training set and validation set partitioning method, and are tested on PDBbind core set (version 2016). Apart from dMaSIF (Sverrisson *et al.* 2021), the predicted results of the other baseline models are derived from their respective published papers. It can be found that our method achieves SOTA performance compared to all baselines.

**Table 1.** Result on PDBbind v.2016 core set.

Model	RMSE ↓	MAE ↓	SD↓	R ↑
**Sequence-based methods**				
DeepDTA	1.443	1.148	1.445	0.749
**Structure-based methods**				
Pafncuy	1.418	1.129	1.375	0.775
OnionNet	1.287	0.983	1.282	0.781
CurvAGN	1.217	0.930	1.191	0.830
**Surface-based method**				
dMaSIF^a^	1.324	1.067	1.277	0.809
**Multimodal methods**				
DPLA	1.255	0.972	1.248	0.820
HaPPy	1.228	0.936	1.221	0.827
**Ours**	**1.151**	**0.882**	**1.138**	**0.851**

a We downloaded the code from the official repository and extended the model for the protein–ligand binding affinity prediction task. For the protein, we use the same surface feature extraction approach as our model. For the ligand and the heterogeneous graph, We use the same approach and parameters as model.

Bold numbers represent the best performance in each metric column, while underlined numbers indicate the second-best performance in each metric column.

After further observation, we find that DeepDTA (Öztürk *et al.* 2018) performs the worst due to relying only on protein sequence information to model molecular graphs and ignoring the spatial structure information of proteins. Pafnucy (Stepniewska-Dziubinska *et al.* 2018) learns the spatial structure of protein–ligand complexes through 3D CNN, but its high-dimensional and sparse 3D matrix data processing results in high computational costs and is sensitive to atomic rotation and translation, affecting prediction accuracy. OnionNet (Zheng *et al.* 2019), which is also based on CNN, has achieved some improvements by incorporating long-range interaction features. However, it does not account for the global structure information of proteins. CurvAGN (Wu *et al.* 2023) adopts a curvature-based adaptive GNN and uses an adaptive graph attention mechanism to integrate geometric structure, long-range molecular interactions and graph heterogeneity to further optimize the representation of protein–ligand complexes. dMaSIF (Sverrisson *et al.* 2021) emphasizes the detailed chemical and geometric features of protein surface pockets and utilizes quasi-geodesic convolutions to learn surface fingerprints. However, these methods focus exclusively on a single modality, ignoring information from other modalities. DPLA (Wang *et al.* 2021b) is a CNN-based regression model that represents protein sequences and binding pockets as two CNN blocks, extracting sequence and structure features through convolution layers. HaPPy (Zhang *et al.* 2023) utilizes pre-trained models to extract protein sequence features and AttentiveFP (Xiong *et al.* 2020) for structure features from protein pocket graphs. However, both methods merely concatenate the extracted features from different modalities, which fails to address the inherent heterogeneity between these modalities.

### 3.5 Ablation studies

**Figure 2.**
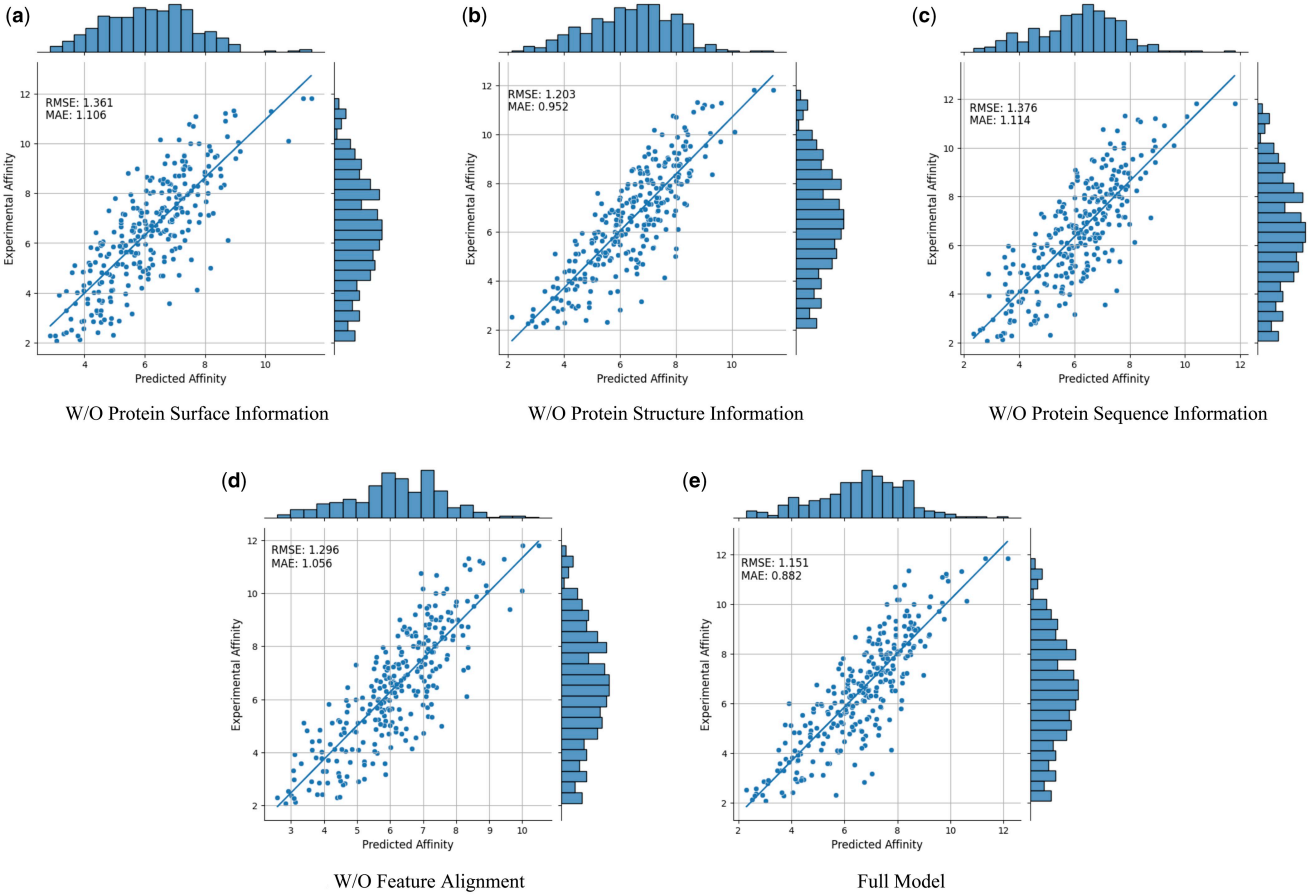
Ablation studies results. (a) W/O Protein Surface Information. (b) W/O Protein Structure Information. (c) W/O Protein Sequence Information. (d) W/O Feature Alignment. (e) Full Model.

In order to further prove the effectiveness and necessity of different modal features and feature alignment, we conduct the following ablation studies: W/O Protein Surface Information, W/O Protein Structure Information, W/O Protein Sequence Information, and W/O Feature Alignment. The results are shown in Table 2 and Fig. 2. The results indicate that when surface information is removed, there is a noticeable decline in performance, demonstrating the critical role of surface information in the model. Similarly, excluding either structure or sequence information leads to a drop in performance, with the elimination of sequence information causing a more significant reduction. This is because sequence information encompasses the global information of the protein, which is vital for the model’s comprehensive understanding of the protein. Furthermore, the model’s performance decreases in the absence of feature alignment. This emphasizes the significance of feature alignment in handling multimodal data, as it helps to lessen the heterogeneity among different modal features, thereby improving the model’s ability to effectively integrate different modal features.

**Table 2.** Ablation studies results.

Model	RMSE ↓	MAE ↓	SD ↓	R ↑
W/O protein surface information	1.361	1.106	1.299	0.801
W/O protein structure information	1.203	0.952	1.173	0.841
W/O protein sequence information	1.376	1.114	1.311	0.797
W/O feature alignment	1.296	1.055	1.198	0.834
**Full model**	**1.151**	**0.882**	**1.138**	**0.851**

Bold numbers represent the best performance in each metric column.

### 3.6 Hyperparameter analysis

**Figure 3.**
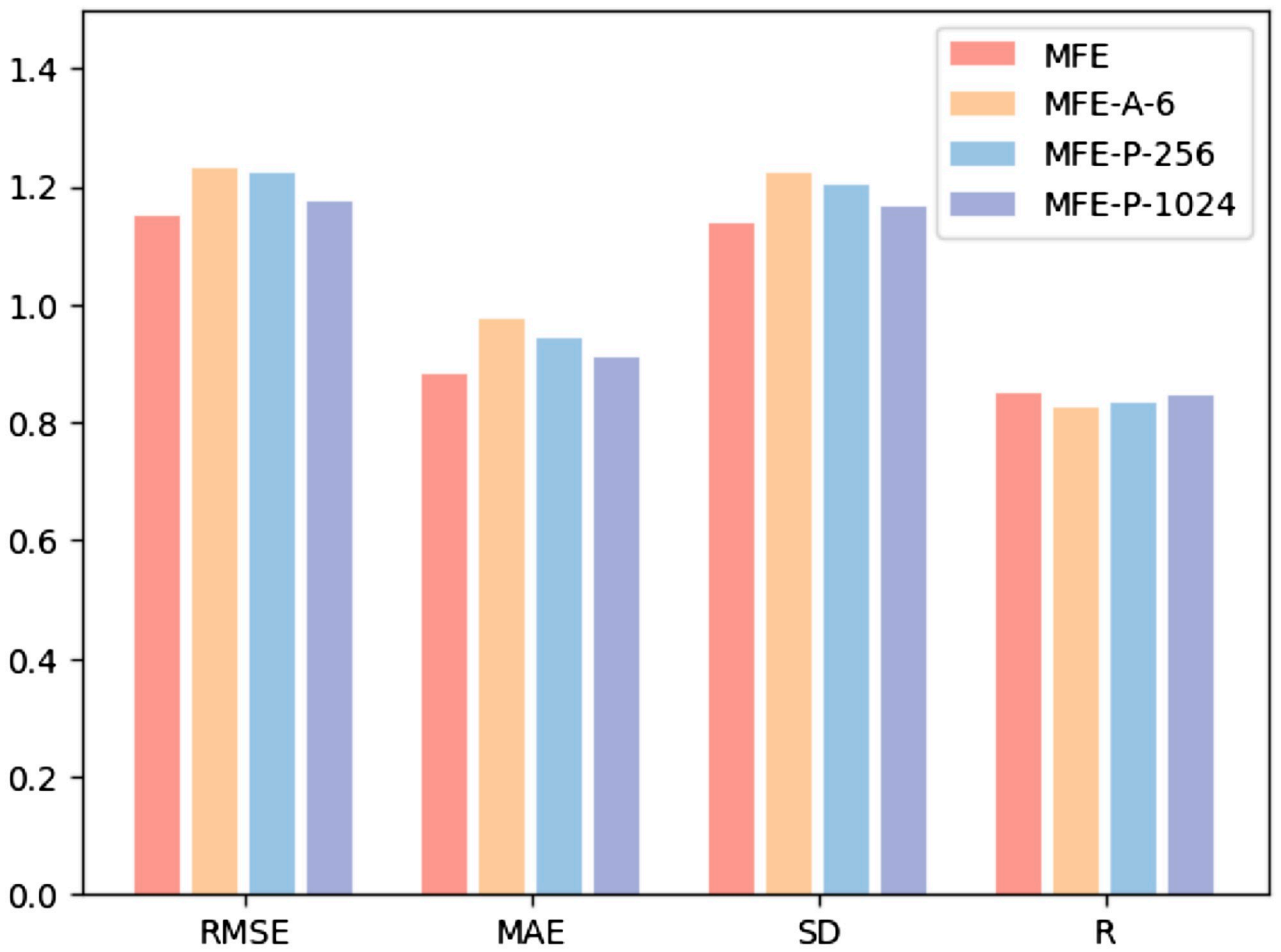
Hyperparameter analysis.

In order to study the impact of different hyperparameters on model performance, we conduct the following three experiments: (i) MFE-A-6: Only 6 basic atom types are used to represent the chemical characteristics of the surface, including hydrogen, carbon, nitrogen, oxygen, phosphorus, sulfur; (ii) MFE-P-256: Select only the 256 surface points closest to the ligand center as protein pocket surface; (iii) MFE-P-1024: Select the 1024 surface points closest to the ligand center as protein pocket surface. Figure 3 shows the results of three different hyperparameter selection methods on the protein–ligand binding affinity prediction task.

**Atom types.** Observing the results in the Fig. 3, we can see that after reducing the atom types input to the model, the overall performance of the model declined, indicating that more atom types can provide richer and multi-dimensional information for the model. In protein–ligand interactions, each atom type carries unique chemical properties that are critical to accurately capture the chemical environment of protein surface pockets because they directly influence the binding site selection and binding affinity of the ligand. By including more atom types, the model is able to more fully understand and simulate the complex chemical features of protein surfaces, leading to better predictions of protein–ligand binding affinity.

**Number of pocket surface points.** Further analysis from the Fig. 3 shows that using fewer surface points to represent protein surface pockets leads to a decline in model prediction accuracy. This is because too few surface points might overlook some crucial environmental features within the protein pockets, affecting the accuracy and reliability of the final binding affinity predictions. On the other hand, using more surface points introduces additional redundant information, causing the model to overly focus on these unimportant details during training and neglect key factors that determine ligand binding characteristics. As a result, the performance of the model also declined.

### 3.7 Feature alignment analysis and visualization

**Figure 4.**
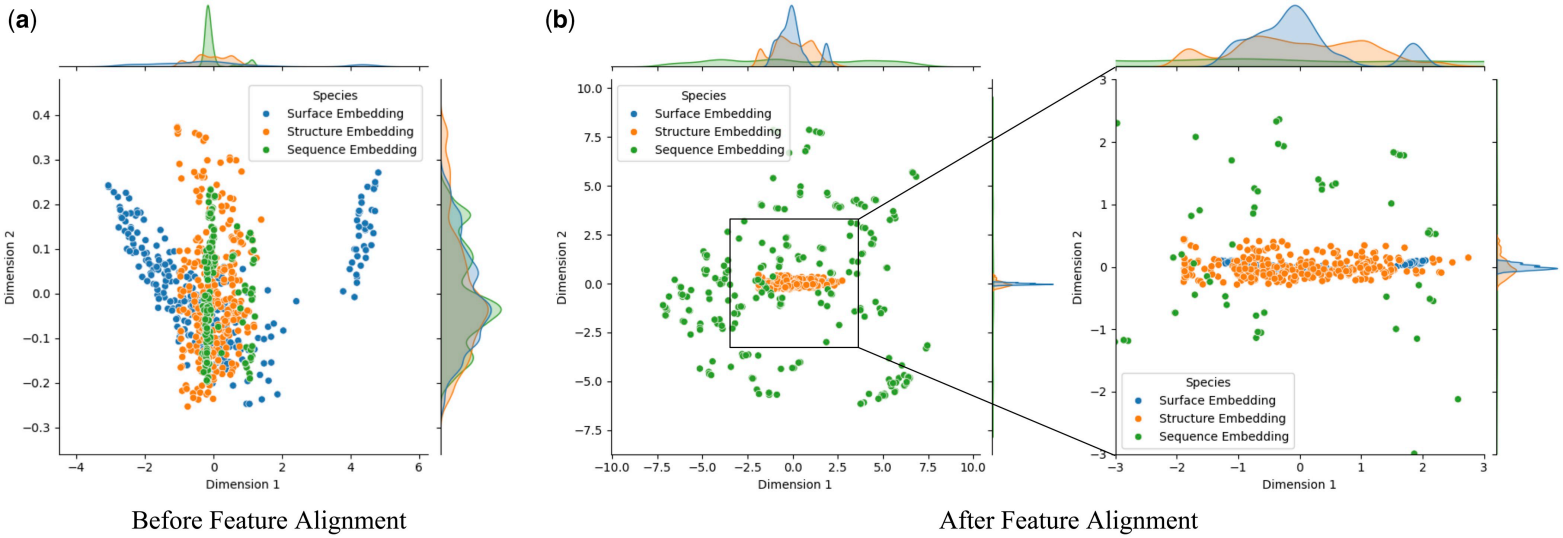
Dimensionality reduction visualization results of protein surface, structure and sequence embedding before feature alignment (a) and after feature alignment (b).

To thoroughly investigate the influence of feature alignment on model performance, we use Principal Component Analysis (PCA) for dimensionality reduction and visual analysis of surface, structure, and sequence features of proteins within the test set. This approach aimed to determine if feature alignment could mitigate heterogeneity among multimodal embeddings. As depicted in Fig. 4, the blue nodes symbolize surface embeddings, the orange nodes denote structure embeddings, and the green nodes signify sequence embeddings. Figure 4a shows the dimensionality reduction visualization before feature alignment, while Fig. 4b shows the result after alignment. These two subfigures allow for a comparison of the distributional differences in various modal embeddings before and after the process of feature alignment.

Analyzing the results of these two subfigures, we can find:

- The visualization before feature alignment (as seen in Fig. 4a) depicts a scattered distribution of nodes across 2D space. Specifically, the structure (orange) embedding and sequence (green) embedding nodes tend to cluster along the Dimension 1 yet display a dispersed arrangement along the Dimension 2. Surface (blue) embedding nodes are more uniformly distributed across both dimensions.
- After the feature alignment process (as shown in Fig. 4b), the nodes across all three categories exhibit a markedly higher concentration within the 2D space. Notably, the surface (blue) and structure (orange) embedding nodes display a substantial increase in spatial density, which suggests a significant enhancement in data feature consistency due to the alignment. Given that the sequence (green) embedding nodes correspond to the embedding of the entire protein sequence and the blue and orange nodes to the protein pocket’s surface and structure embedding, respectively, the green points remain more diffuse after feature alignment. Nevertheless, they show a propensity to aggregate toward the central region. Therefore, the analysis demonstrate that feature alignment can enhance the model’s capability to process and fuse multimodal data.

Therefore, feature alignment significantly enhances the coherence between the embeddings of protein surface, structure, and sequence. This is due to the optimization of multimodal feature interactions within the Transformer through attention mechanisms, which calculate attention weights between different features. This enhances the model’s ability to capture key information, allowing data from different modalities to aggregate more closely in the feature space, thereby reducing noise and errors when the model identifies protein–ligand interactions.

## 4 Conclusion

In this work, we propose a novel framework that unifies the information from protein surfaces, 3D structures and sequences, and use Transformer to align features of different modalities. We then evaluate our model on the Protein–ligand binding affinity prediction task, demonstrating the effectiveness of the model. The final ablation study as well as feature alignment analysis demonstrate the importance of each component in our framework. In summary, by studying the surfaces of proteins, we can gain a deeper understanding of how proteins interact with other biomolecules. In our future work, we will explore the protein surfaces more thoroughly to uncover their broader applications in bioinformatics.

## Supplementary Material

btae413_Supplementary_Data

## Data Availability

The source code and data are available at https://github.com/Sultans0fSwing/MFE.
