# Supplementary material for: Surface-based multimodal protein–ligand binding affinity prediction
Source: Bioinformatics. 2024 Jun 21;40(7):btae413. doi: 10.1093/bioinformatics/btae413 (PMC11629684; doi:10.1093/bioinformatics/btae413)
Supplement: btae413_Supplementary_Data [file btae413_supplementary_data.pdf]

Supplementary Data For Paper: Surface-based Multimodal Protein-Ligand Binding Affinity Prediction

1 Protein Surface

1.1 Surface generation

1.1.1 Fast sampling

Our data is sourced from PDBBind dataset (version 2016) (Wang et al., 2005), where protein structures typically consist of A = 3,000 - 15,000 atoms. To model the protein surface, we use a cloud of atoms as input and employ a smooth distance function or metaball to describe the protein surface (Figure 1). Initially, around the neighborhood of a specific atom, 20 points are generated from a Gaussian random distribution based on the atom's smooth distance function:

$$SDF(x) = -\sigma(x) \cdot log \sum_{k=1}^{A} exp(- \parallel x - a_k \parallel / \sigma_k) \tag{1}$$

$$\sigma(x) = \frac{\sum_{k=1}^{A} exp(- \parallel x - a_k \parallel) \sigma_k}{\sum_{k=1}^{A} exp(- \parallel x - a_k \parallel)} \tag{2}$$

where σ_k is atomic radius to each atom a_k and $\sigma(x)$ is the average atomic radius in a neighborhood of point x.

Subsequently, these sampling points are adjusted at a radius of r = 1.05 Å by minimizing the squared loss function (Eq.(3)) through the gradient descent method, executing four gradient steps with a learning rate of 1:

$$E(x_1, ..., x_N) = \frac{1}{2} \sum_{i=1}^{N} (SDF(x_i) - r)^2 \tag{3}$$

Finally, the points are screened to retain those whose distance values fall within the range of $r - 0.1$ Å to $r + 0.1$ Å, and which show an increment greater than 0.5 Å after four consecutive gradient descent steps of size 1 Å. We then placed all points into cubic bins with a side length of 1 Å, keeping one average sample per bin to ensure uniform density in our sampling. After sampling the whole protein surface, we then selected the N = 512 surface points closest to the center of the ligand as surface pockets.

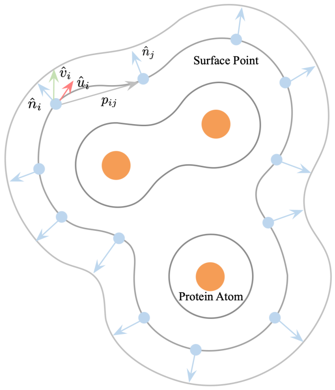

Figure 1: Surface point cloud featurization. Level sets (gray) surrounding the atoms (orange) are utilized to find surface points (blue). The vector $\hat{n}_i$ represents the unit normal vector at each point, while $(\hat{u}_i, \hat{v}_i)$ denote the orthogonal tangent vectors in the local coordinate system.

1.1.2 Construction of local coordinate system

For each sample point $x_i (i = 1, 2, ..., N)$, we normalize the gradient of the distance function to obtain the unit normal vector $\hat{n}_i$. To estimate a local coordinate system $(\hat{n}_i, \hat{u}_i, \hat{v}_i)$, we smooth this vector field using a Gaussian kernel with $\sigma = 9$ Å, calculated as follows:

$$\hat{n}_i \leftarrow Normalize(\sum_{j=1}^{N} exp(-\left\|x_i - x_j\right\|^2 / 2\sigma^2)\hat{n}_j) \tag{4}$$

Then, let $\hat{n}_i = [x, y, z]$, we can calculate the tangential vectors $\hat{u}_i, \hat{v}_i$ using the following equations:

$$\hat{u}_i = [1 + sax^2, sb, -sx], \hat{v}_i = [b, s + ay^2, -y] \tag{5}$$

where $s = sign(z), a = -1/(s + z), b = axy$.

1.1.3 Chemical feature

For each point x_i, we first find the 16 nearest atomic centers $\{a_1^i, ..., a_{16}^i\}$, which have types $\{t_1^i, ..., t_{16}^i\}$ encoded into a one-hot encoded vector in R^{22}. The vectors $[t_k^i, 1/||x_i - a_k^i||] \in R^{23}$ are processed by a multi-layer perceptron (MLP) to generate feature vectors $C_{i,k} \in R^{22}$. These feature vectors $C_{i,k}$, for $k = 1, 2, 3, ..., 16$, are then aggregated through summation. Subsequently, a second MLP linearly maps this sum to a chemical feature vector $C_i \in R^{22}$.

1.2 Quasi-geodesic convolutions on point clouds

1.2.1 Work on oriented point clouds

Since calculating the geodesic distance between each pair of points requires a large overhead, using the following formula to approximate the geodesic distance can help simplify the calculation process while maintaining the basic structural characteristics of the data:

$$d_{ij} = \| x_i - x_j \| (2 - < (\hat{n}_i), (\hat{n}_j) >) \tag{6}$$

A smooth Gaussian window of $\sigma = 9$ Å is then used as a filter for the geodesic distance, which is defined as:

$$w(d_{ij}) = exp(-\frac{d_{ij}^2}{2\sigma^2}) \tag{7}$$

The relative position and orientation of a protein surface point x_i and its neighboring point x_j in the local coordinate system $(\hat{n}_i, \hat{u}_i, \hat{v}_i)$ can be encoded as two 3D vectors:

$$p_{ij} = [p_{ij}^{\hat{n}}, p_{ij}^{\hat{u}}, p_{ij}^{\hat{v}}] = [(x_j - x_i)^T] \cdot [\hat{n}_i|\hat{u}_i|\hat{v}_i] \tag{8}$$

$$q_{ij} = [q_{ij}^{\hat{n}}, q_{ij}^{\hat{u}}, q_{ij}^{\hat{v}}] = [(\hat{n}_j - \hat{n}_i)^T] \cdot [\hat{n}_i|\hat{u}_i|\hat{v}_i] \tag{9}$$

For the sake of computational efficiency, we use MLPs as trainable filters.

1.2.2 Local orientation and curvatures

To resolve the rotational freedom of tangent vectors $(\hat{u}_i, \hat{v}_i)$ within the tangent plane at a low computation cost, the first tangent vector $\hat{u}_i = \hat{u}(x_i)$ is aligned with the geometric gradient $\bigtriangledown^{\hat{u},\hat{v}} P(x_i)$ of a trainable potential $P(x_i) = P_i = MLP(f_i)$. We then use quasi-geodesic convolutions to approximate the gradients by utilizing the derivative of a Gaussian filter on the tangent plane:

$$\bigtriangledown P(x_i) \simeq \frac{1}{N} \sum_{j=1}^{N} w(d_{ij})[p_{ij}^{\hat{u}}, p_{ij}^{\hat{v}}] P_j \in R^2 \tag{10}$$

The tangent basis $(\hat{u}_i, \hat{v}_i)$ is updated using the standard trigonometric formulae.

To compute the local curvatures, we use quasi-geodesic convolutions with Gaussian windows of radii $\sigma \in [1, 2, 3, 5, 10]$ Å and quadratic filter functions. These functions estimate the local covariances $Cov_{\sigma,i}^{\hat{u},\hat{v}}(p, p)$ and $Cov_{\sigma,i}^{\hat{u},\hat{v}}(p, q)$ of the point positions and normals as 2×2 matrices in the tangent plane $(\hat{u}_i, \hat{v}_i)$:

$$p = [p_{ij}^{\hat{u}}, p_{ij}^{\hat{v}}] = [(x_j - x_i)^T] \cdot [\hat{u}_i|\hat{v}_i], q = [q_{ij}^{\hat{u}}, q_{ij}^{\hat{v}}] = [(\hat{n}_j - \hat{n}_i)^T] \cdot [\hat{u}_i|\hat{v}_i] \tag{11}$$

The 2×2 shape operator at point x_i and scale σ is then approximated as follows:

$$S_{\sigma,i} = (\lambda^2 Id_{2\times2} + Cov_{\sigma,i}^{\hat{u},\hat{v}}(p, p))^{-1} Cov_{\sigma,i}^{\hat{u},\hat{v}}(p, q) \tag{12}$$

where regularization parameter $\lambda = 0.1$ Å.

The Gaussian curvature $K_{\sigma,i}$ and mean curvature $H_{\sigma,i}$ at scale σ are defined as follows:

$$K_{\sigma,i} = det(S_{\sigma,i}), H_{\sigma,i} = trace(S_{\sigma,i}) \tag{13}$$

Then, we concatenate the chemical features $C_i \in R^{22}$ with the Gaussian curvature $K_i \in R^5$ and mean curvature $H_i \in R^5$ to create a full feature vector $f_i \in R^{32}$. The initial features of protein surface point clouds are shown in Table 1.

Table 1: The initial features of protein surface point clouds

Type	Count	Descriptions
**Chemical feature**		
atom types	22	One-hot coding for atom types (H, Li, C, N, O, Na, Mg, P, S,K, Ca, Mn, Fe, Co, Ni, Cu, Zn, Se, Sr, Cd, Cs, Hg)
**Geometric feature**		
Mean curvature	5	Computed at 5 scales ranging from 1 Å to 10 Å
Gaussian curvature	5	Computed at 5 scales ranging from 1 Å to 10 Å

1.2.3 Trainable convolutions

To update the feature vector f_i, we employ quasi-geodesic convolutions on the molecular surface, which rely on a trainable MLP to transform the input feature $f_i \in R^{32}$ into the output feature $f_i' \in R^{128}$ according to the following equation:

$$f_i' \leftarrow \sum_{j=1}^{N} w(d_{ij}) MLP(p_{ij}) f_j \tag{14}$$

2 GVP Module

The GVP (Jing et al., 2021) takes scalar features $s \in R^n$ and vector features $V \in R^{v \times 3}$ as inputs, and transforms them into new scalar features $s' \in R^m$ and vector features $V' \in R^{\mu \times 3}$. The transformation equations are as follows:

(1) Scalar Feature Transformation:

$$s' = \sigma((concat(s, \|V_h\|_2) W_m + b \tag{15}$$

where $concat(s, \|V_h\|_2)$ is the concatenation of the scalar features and the L_2 norm of the transformed vector features V_h, $W_m \in R^{(n+h) \times m}$ is the weight matrix, and b is the bias vector. The function σ is an activation function.

(2) Vector Feature Transformation:

$$V' = \sigma^+(\|V_h W_\mu\|_2) \odot V_h W_\mu \tag{16}$$

Here, $V_h = V W_h$ with $W_h \in R^{v \times h}$ being a weight matrix that projects the original vector features into a higher-dimensional space. $W_\mu \in R^{h \times \mu}$ is another weight matrix transforming V_h to the new vector feature dimension. The operator $\odot$ denotes element-wise multiplication, and σ^+ is an activation function specifically suitable for vectors, ensuring that the transformed vectors maintain appropriate directional properties.

3 Multimodal Feature Alignment Module

Table 2: Parameter details of multimodal feature alignment module

Component	Description	Size
Embedding Size	Dimensionality of embeddings for each modality	Surface embedding: 128, Structure embedding: 128, Sequence embedding: 128
Encoder Settings	Specific configurations of encoder	Input size=128, Output size=128, Multi-head attention heads=8, Feed-forward size=256, Dropout rate=0.3, Number of Layers=1
Decoder Settings	Specific configurations of decoder	Input size=128, Output size=128, Multi-head attention heads=8, Feed-forward size=256, Dropout rate=0.3, Number of Layers=1

4 Heterogeneous Graph

All node and edge features used in the heterogeneous graph are listed in Table 3 and Table 4, respectively.

Table 3: Node features of heterogeneous graph

Node feature	Count	Descriptions
atom types	9	one-hot coding for atom types (B, C, N, S, O, P, Se, Halogen, Metal)
Properties	9	Degree, Charge, Part of a ring?, Is aromatic?

Table 4: Edge features of heterogeneous graph

Edge feature	Count	Descriptions
Distance	1	Euclidean distance between two nodes
Angle statistics	3	The maximum, sum, and average scaled angle between neighboring nodes in 3D space.
Area statistics	3	The maximum, sum, and average areas between neighboring nodes in 3D space.
Distance statistics	3	The maximum, sum, and average scaled distances between neighboring nodes in 3D space.
IsAromatic	1	Is the bond aromatic?
IsRing	1	Is the bond part of a ring?

In a heterogeneous graph $G = (V_l, V_p, E)$, nodes in different subsets possess distinct attributes and feature vector lengths, necessitating separate message-passing operations for each group. For each node u and v, the node states are updated through an aggregation function AGG and an update function U as follows:

$$h_u^k = U_u\left(h_u^{k-1}, AGG\left(M_u\left(h_v^{k-1}, h_u^{k-1}, e_{uv}\right)\right)\right), \quad v \in N(u) \tag{17}$$

$$h_v^k = U_v\left(h_v^{k-1}, AGG\left(M_v\left(h_u^{k-1}, h_v^{k-1}, e_{vu}\right)\right)\right), \quad u \in N(v) \tag{18}$$

Here, $N(u)$ and $N(v)$ denote the sets of neighbor nodes for u and v, respectively.

The initial feature vector of each edge, e_{init}, represents the distance between the two connected nodes. This vector is processed through a linear layer L and then concatenated with the feature vectors of the two corresponding nodes:

$$m_{pl} = MLP(concat((h_u, h_v, L(e_{init})))) \tag{19}$$

Following this, the processed features m_{pl} are subjected to max pooling and weighted sum pooling:

$$m_{max} = MaxPooling(m_{pl}) \tag{20}$$

$$m_w = \sum_{pl \in E} \tanh(W_2 m_{pl}) \times m_{pl} \tag{21}$$

At last, We combine them into a global embedding vector (m_w, m_{max}) for the entire heterogeneous graph.

5 Error Evaluation Metrics

In this part, we provide the mathematical expressions for the evaluation metrics referenced in the experiments:

(1) Root Mean Squared Error (RMSE):

$$RMSE = \sqrt{\frac{1}{N}\sum_{i=1}^{N}(y_i - \hat{y}_i)^2}, \tag{22}$$

(2) Mean Absolute Error (MAE):

$$MAE = \frac{1}{N}\sum_{i=1}^{N}|y_i - \hat{y}_i|, \tag{23}$$

(3) Standard Deviation (SD):

$$SD = \sqrt{\frac{1}{N-1}\sum_{i=1}^{N}[y_i - (a\hat{y}_i + b)]^2}, \tag{24}$$

(4) Pearson Correlation Coefficient (R):

$$R = \frac{\sum_{i=1}^{N}(\hat{y}_i - \bar{\hat{y}})(y_i - \bar{y})}{\sqrt{\sum_{i=1}^{N}(\hat{y}_i - \bar{\hat{y}})^2(y_i - \bar{y})^2}}. \tag{25}$$

Among them, N represents the number of sample pairs used in the experiment, y_i and $\hat{y}_i$ represent the true value and predicted value of the binding affinity of the i-th sample pair respectively. The a and b are the slope and intercept of the regression line, respectively.

6 Case Study

To evaluate our model's performance on docked complexes, we conducted the following case study: We selected 5 different types of protein-ligand complexes from the 285 compounds in the PDBbind core set (version 2016) . We redocked these using Surflex-Dock (Jain, 2007) in '-pgeom' mode, generating up to 20 poses per ligand. We then chose the top three poses with the lowest RMSD for affinity prediction. The experimental results are presented in Table 5.

Table 5: Performance evaluation of docked protein-ligand complexes

PDB ID	Experimental Affinity	Model Prediction	Docking P1 Prediction	Docking P2 Prediction	Docking P3 Prediction
2v00	3.66	3.42	2.86	2.73	2.78
3prs	7.82	7.93	7.90	7.36	7.28
3qgy	7.80	7.85	7.94	7.86	7.79
4f3c	11.82	11.69	10.63	10.45	10.66
4m0y	6.46	6.64	6.91	6.90	6.98

- PDB ID: Identifier for each protein-ligand complex.
- Experimental Affinity: The experimentally measured binding constant, expressed in terms of -logKd/Ki, used to validate the accuracy of the model's predictions.
- Model Prediction: Predictions made by the model based on the experimentally determined docking pose.
- Docking P1-3 Prediction: Represents the model's predictions based on different docking poses obtained during the docking process. This highlights how variations in docking pose can affect prediction accuracy.

References

A. N. Jain. Surflex-dock 2.1: robust performance from ligand energetic modeling, ring flexibility, and knowledge-based search. *Journal of computer-aided molecular design*, 21: 281–306, 2007.

B. Jing, S. Eismann, P. Suriana, R. J. L. Townshend, and R. Dror. Learning from protein structure with geometric vector perceptrons. 2021.

R. Wang, X. Fang, Y. Lu, C.-Y. Yang, and S. Wang. The pdbbind database: Methodologies and updates. *Journal of Medicinal Chemistry*, page 4111–4119, Jun 2005.
